# The Vicious Cycle of Sleep Disruption and Inflammaging: Mechanisms, Consequences, and Therapeutic Opportunities

**DOI:** 10.1111/acel.70798

**Published:** 2026-11-04

**Authors:** Fu‐Ying Zhao, Jia‐Mei Wang, Shi‐Yang Lu, Liang Hao, Hai‐Yan Zhang, Hua‐Qin Wang

**Affiliations:** ^1^ Department of Biochemistry & Molecular Biology China Medical University Shenyang Liaoning Province China; ^2^ Department of Laboratory Medicine The First Hospital of China Medical University Shenyang Liaoning Province China; ^3^ Department of Chemistry, School of Forensic Medicine China Medical University Shenyang China; ^4^ Department of Geriatrics The First Affiliated Hospital of China Medical University Shenyang Liaoning Province China

**Keywords:** aging, cellular senescence, circadian rhythm, immunosenescence, obstructive sleep apnea, sleep disruption

## Abstract

Aging is characterized by two interdependent hallmarks: a chronic low‐grade inflammatory state known as inflammaging, and a progressive deterioration of sleep and circadian rhythms. Emerging evidence indicates that these are not merely parallel processes but are mechanistically intertwined in a self‐perpetuating cycle. This review synthesizes current knowledge on how age‐related sleep and circadian disturbances exacerbate inflammaging through distinct molecular and cellular pathways. We examine the nocturnal dysregulation of the immune system, focusing on innate immune hyperactivation (e.g., via NLRP3 inflammasome and TLR4 signaling), cellular senescence (SASP), adaptive immune decline (T‐cell aging), and disruption of systemic pathways such as the gut‐brain axis. Clinical and epidemiological data link sleep disorders, particularly obstructive sleep apnea, to elevated inflammatory markers, accelerated epigenetic aging, and cognitive decline. Conversely, inflammaging impairs circadian regulatory structures, further degrading sleep quality. Interventions targeting the sleep‐immune axis, including melatonin, circadian reinforcement, senotherapeutics, and lifestyle modifications, hold promise for mitigating inflammaging. We conclude that sleep disruption is not a passive symptom but an active driver of immunological aging, and propose that targeting this axis represents a viable strategy for promoting healthy longevity.

## Graphical abstract

Sleep disruption and circadian decline drive inflammaging and immune aging via the gut‐brain axis, NLRP3, and TLR4, forming a vicious cycle that accelerates cognitive decline and epigenetic aging. Targeting this sleep‐immune axis offers a viable strategy for promoting healthy longevity.

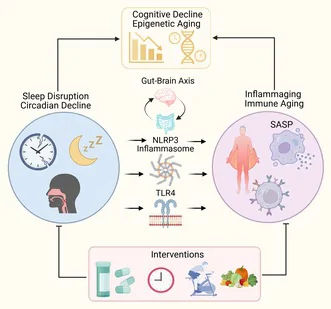

## 1 Introduction

Aging is marked by two interrelated hallmarks: a chronic low‐grade inflammatory state termed inflammaging and progressive deterioration of sleep architecture and circadian regulation (Franceschi et al. 2018; Straub 2017; Muller and Di Benedetto 2025; Segura‐Collar et al. 2025; Keil et al. 2023). Inflammaging, arising from immune‐metabolic dysregulation, is a pivotal contributor to neurodegenerative, cardiovascular, and metabolic diseases. Aging also predictably alters sleep, reducing slow‐wave sleep, and circadian rhythmicity while increasing fragmentation (Fonken et al. 2016; Lo et al. 2014; Zanders et al. 2026; Muller and Di Benedetto 2025; Xu et al. 2025).

Accumulating evidence indicates these processes are not merely concurrent but mechanistically linked. Sleep actively supports immune homeostasis (Simpson and Dinges 2007), its disruption may therefore transcend being a passive symptom of aging to become an active catalyst of the inflammatory cascade. Epidemiological and clinical data link poor sleep quality and disorders such as obstructive sleep apnea (OSA) to accelerated biological aging (as evidenced by telomere shortening) and elevated systemic inflammatory markers (Atienza et al. 2018; Tempaku et al. 2025). This raises a central unresolved question: is sleep disruption a primary driver of inflammaging, a consequence thereof, or, most plausibly, a critical component of a self‐reinforcing vicious cycle that accelerates immunological decline (Cardinali et al. 2022; Nagayach et al. 2025)? Our proposed sleep‐immune‐inflammaging axis addresses this by positioning sleep disruption at the center of this dynamic, not as a passive bystander but as an active, modifiable driver of the aging immune landscape.

Several frameworks have been proposed to explain aging‐immunity‐metabolism. The neuro‐immune senescence axis emphasizes bidirectional communication between the central nervous system (particularly the hypothalamic–pituitary–adrenal axis and sympathetic nervous system) and immune cells, highlighting how age‐related neuroendocrine changes (e.g., cortisol dysregulation) perpetuate systemic inflammation (Straub 2017; Bentivoglio et al. 2006; Gonzalez‐Hurtado et al. 2025). Seminal work by Miller and colleagues mapped the molecular pathways linking stress, circadian disruption, and cellular senescence (Miller and Raison 2016; Irwin and Opp 2017; Nie et al. 2025). The metaflammation hypothesis, articulated by Franceschi and colleagues, posits that metabolic surplus and nutrient‐sensing pathways (e.g., mTOR, AMPK) drive a chronic low‐grade inflammation underlying age‐related diseases (Franceschi et al. 2018). These models have advanced the field but largely treat sleep and circadian disruption as downstream consequences or peripheral contributors, rather than primary drivers or central organizing nodes of inflammatory aging.

Here, we propose a distinct sleep‐immune‐inflammaging axis that positions sleep disruption as a factor of immunological aging. This model offers several unique features: (i) it identifies sleep architecture (slow‐wave sleep loss, fragmentation) and circadian misalignment as primary instigators of specific molecular cascades (NLRP3 inflammasome activation, SASP induction, T‐cell senescence); (ii) it integrates recently discovered sleep‐dependent systemic amplifiers (the gut‐brain axis and the glymphatic system) that transform peripheral immune disturbances into sustained neuroinflammation; and (iii) it emphasizes that sleep is not merely a passive state but a critical window for immune homeostasis and waste clearance, making it a uniquely tractable intervention target. Unlike less accessible axes (e.g., altering central neuroendocrine set‐points), sleep quality and circadian rhythms can be modified through behavioral, environmental, and pharmacological interventions, offering a practical entry point to disrupt the vicious cycle of inflammaging. By framing sleep as the linchpin connecting neuroendocrine, metabolic, and immune aging, our model provides a comprehensive, actionable framework for future research and therapeutic development.

This review dissects the nocturnal dysregulation of the immune system. We analyze how age‐related sleep and circadian disturbances leverage specific molecular and cellular pathways to fuel inflammaging and evaluate the therapeutic promise of targeting the sleep‐immune axis to promote healthy aging.

### 1.1 Review Methodology, Definitions, and Scope

We searched PubMed/MEDLINE, Web of Science, Scopus, Embase, and Google Scholar from database inception to August 2026 using terms combining sleep disruption, sleep fragmentation, insomnia, obstructive sleep apnea, circadian rhythm, immunosenescence, inflammaging, cellular senescence, SASP, NLRP3, gut–brain axis, and glymphatic. We included peer‐reviewed original research, systematic reviews, meta‐analyses, and clinical guidelines in English, excluding conference abstracts, case reports, and non‐peer‐reviewed literature. Animal studies provided mechanistic insight; human studies were prioritized for clinical claims. No date restriction was applied, but recent literature (2018–2026) was emphasized.

We use “sleep disruption” as an umbrella term for: (i) insomnia disorder (DSM‐5/ICSD‐3 criteria: difficulty initiating or maintaining sleep, or early morning awakening, with daytime impairment ≥ 3 nights/week for ≥ 3 months); (ii) OSA (AHI/RDI ≥ 5 with symptoms or ≥ 15 regardless of symptoms, or physician diagnosis); (iii) sleep fragmentation or abnormal duration (short < 6 h; long > 9 h); (iv) circadian rhythm sleep–wake disorders, including delayed sleep–wake phase disorder (DSWPD) and shift work disorder; and (v) poor subjective sleep quality (e.g., Pittsburgh Sleep Quality Index > 5). Self‐reported and objective measures (polysomnography, actigraphy) are distinguished.

OSA is a peripheral, mechanically driven disorder strongly associated with overweight/obesity, although not confined to it. In cited studies, OSA was generally defined by AHI/RDI thresholds or clinical diagnosis; where self‐report or risk questionnaires (e.g., STOP‐BANG) replaced objective AHI, this is noted as a limitation. Excessive daytime sleepiness and CPAP or CBT‐I use are important moderators; studies not accounting for them may overestimate sleep‐specific immune effects.

We define aging as the biological process from midlife onward, with a clinical focus on 45–75 years; mechanistic evidence derives largely from animal models. Sleep disruption and inflammaging are not universal features of aging. Their prevalence varies by population, sex, socioeconomic status, comorbidity, and environmental exposures. This model applies to individuals with chronic sleep and/or circadian disruption, not all older adults. Where evidence derives from selected clinical populations (e.g., OSA clinics), this is stated to avoid overgeneralization.

### 1.2 Graphical Abstract/Conceptual Framework

The dynamic interplay between sleep disruption, circadian dysregulation, immune dysfunction, and inflammaging constitutes a self‐perpetuating cycle. Age‐related degradation of central (e.g., suprachiasmatic nucleus) and peripheral (e.g., immune cell) clocks leads to sleep fragmentation and loss of rhythmicity. This disruption, epitomized by conditions like obstructive sleep apnea, directly incites innate immune hyperactivation (via pathways such as NLRP3 and TLR4), promotes cellular senescence and the senescence‐associated secretory phenotype (SASP), and impairs adaptive immunity (e.g., T‐cell aging). Furthermore, it compromises systemic barriers, notably the gut‐brain axis, increasing intestinal permeability and systemic inflammation. The resulting pro‐inflammatory milieu (elevated IL‐6, TNF, CRP) subsequently inflicts damage on circadian structures and sleep‐regulatory brain regions, thereby reinforcing the cycle and accelerating overarching biological aging processes. To encapsulate this bidirectional relationship, we propose a conceptual model (Figure 1) illustrating how sleep and circadian disturbances interact with immune dysfunction to fuel a vicious cycle of inflammaging.

**FIGURE 1:**
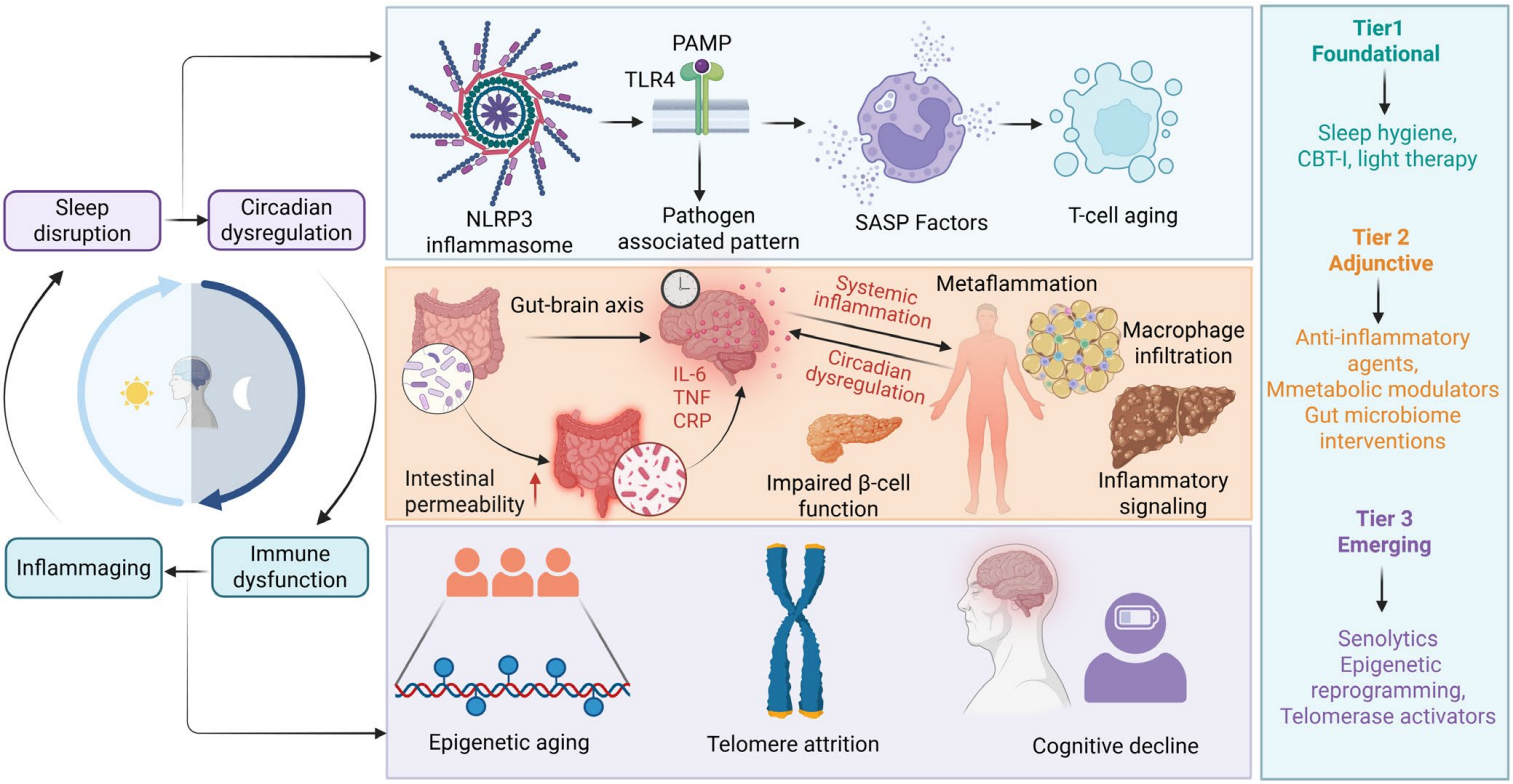
The sleep–immune–inflammaging axis: A self‐perpetuating vicious cycle. Sleep disruption, driven by age‐related circadian deterioration, occupies a central linchpin position, actively driving immunological aging through a hierarchical cascade: (i) direct immunosenescence (innate hyperactivation via NLRP3/TLR4, SASP induction, T‐cell aging); (ii) systemic amplification via gut–brain axis dysfunction and metaflammation; and (iii) collapse of glymphatic clearance, allowing accumulation of pro‐inflammatory mediators. The resulting systemic inflammation feeds back to impair circadian structures and sleep‐regulatory brain regions, closing a feed‐forward loop that accelerates epigenetic aging, telomere attrition, and cognitive decline. Foundational (Tier 1), adjunctive (Tier 2), and emerging (Tier 3) interventions target distinct nodes in this cycle, offering a roadmap for precision sleep medicine.

## 2 Fundamentals of the Sleep‐Immune Axis: Hierarchical Circadian Orchestration

At all ages, sleep is an active neurological and physiological state essential for immune homeostasis. In healthy young adults, sleep efficiently supports immunoregulation and waste clearance, yet youth does not safeguard against metabolic and immune dysregulation: childhood and adolescent obesity, early metabolic dysfunction, and subclinical inflammation are increasingly prevalent and may set the stage for later inflammaging. Thus, the sleep‐immune axis is therefore a lifelong regulator whose resilience declines with age but is already challenged in younger populations. This coordination is governed by a hierarchically organized circadian system that temporally aligns immune functions across central, neuroendocrine, and cellular levels (Acuna‐Castroviejo et al. 2017).

The master pacemaker in the suprachiasmatic nucleus (SCN) provides top‐down regulation, synchronizing peripheral oscillators within immune tissues. A principal neuroendocrine arm is nocturnal melatonin secretion. Beyond its soporific effects, melatonin is a pleiotropic with potent anti‐inflammatory and antioxidant properties, regulating key metabolic and defense pathways that involve sirtuins (Cardinali 2021; Hardeland 2019; Hardeland et al. 2011). Age‐related decline in melatonin production is thus critical to the erosion of rhythmic immunoregulation and neuroprotection (Bocheva et al. 2024).

This central command integrates with cell‐intrinsic circadian clocks within the immune system itself. For instance, the core clock gene *Bmal1* drives diurnal oscillations in neutrophil aging and trafficking, a finely tuned program balancing antimicrobial efficacy against vascular integrity (Adrover et al. 2019). More broadly, leukocyte trafficking between blood and tissues exhibits robust circadian patterns that optimize immune surveillance and effector responses (Esquifino et al. 2007; Nathan et al. 2021).

Notably, these rhythmic trafficking patterns are not immutable. Aging dampens the amplitude of leukocyte migratory rhythms, contributing to the persistent low‐grade inflammation and impaired immune surveillance in inflammaging (Nathan et al. 2021). This age‐related loss of temporal coordination sets the stage for the innate hyperactivation and adaptive decline discussed in Section 3.

Consequently, sleep in a young healthy system orchestrates predictable immune cell redistribution and favors an anti‐inflammatory cytokine milieu. Behavioral sleep, rhythmic neuroendocrine signals (e.g., melatonin, cortisol), and autonomous cellular clocks establishes a foundational temporal program for immune resilience. However, a critical gap persists: although slow‐wave sleep is hypothesized to consolidate adaptive immune memory, direct mechanistic evidence in humans remains sparse and represents a vital research frontier.

An intriguing but unexplored question is whether sleep and circadian rhythms modulate trained immunity, the long‐term reprogramming of innate immune cells by prior insults (Netea et al. 2016). Because trained immunity involves metabolic and epigenetic reprogramming pathways tightly linked to the circadian clock (e.g., mTOR, HIF‐1α), sleep disruption may not only trigger acute inflammation but also permanently rewire innate immune system toward a hyperresponsive, pro‐inflammatory state (Cheng et al. 2014; Netea et al. 2016; McAlpine et al. 2022; Touw et al. 2026). This represents a critical frontier because it could explain the persistent, self‐perpetuating nature of inflammaging even after sleep restoration.

This finely orchestrated temporal program represents the youthful, resilient state of the sleep‐immune axis, in which behavioral sleep, rhythmic neuroendocrine signals, and cellular clocks synergize to maintain immune homeostasis (Simpson and Dinges 2007; Nathanson 2021). However, as detailed below, aging progressively erodes each layer of this hierarchy, from SCN integrity to immune cell‐intrinsic clocks, transforming this protective axis into a vicious driver of inflammaging. Understanding this foundational architecture is essential to appreciate how its disruption unleashes the pathological cascades described below.

## 3 How Sleep Disruption Exacerbates Inflammaging: Mechanistic Insights

Sleep disruption acts as a potent catalyst for inflammaging through a hierarchical, interconnected network of molecular and cellular pathways that converge to accelerate immune senescence and establish a self‐sustaining pro‐inflammatory state. We first examine direct effects on immune cell populations, then explore systemic amplifiers, and finally identify circadian disruption as the overarching driver (Figure 2).

**FIGURE 2:**
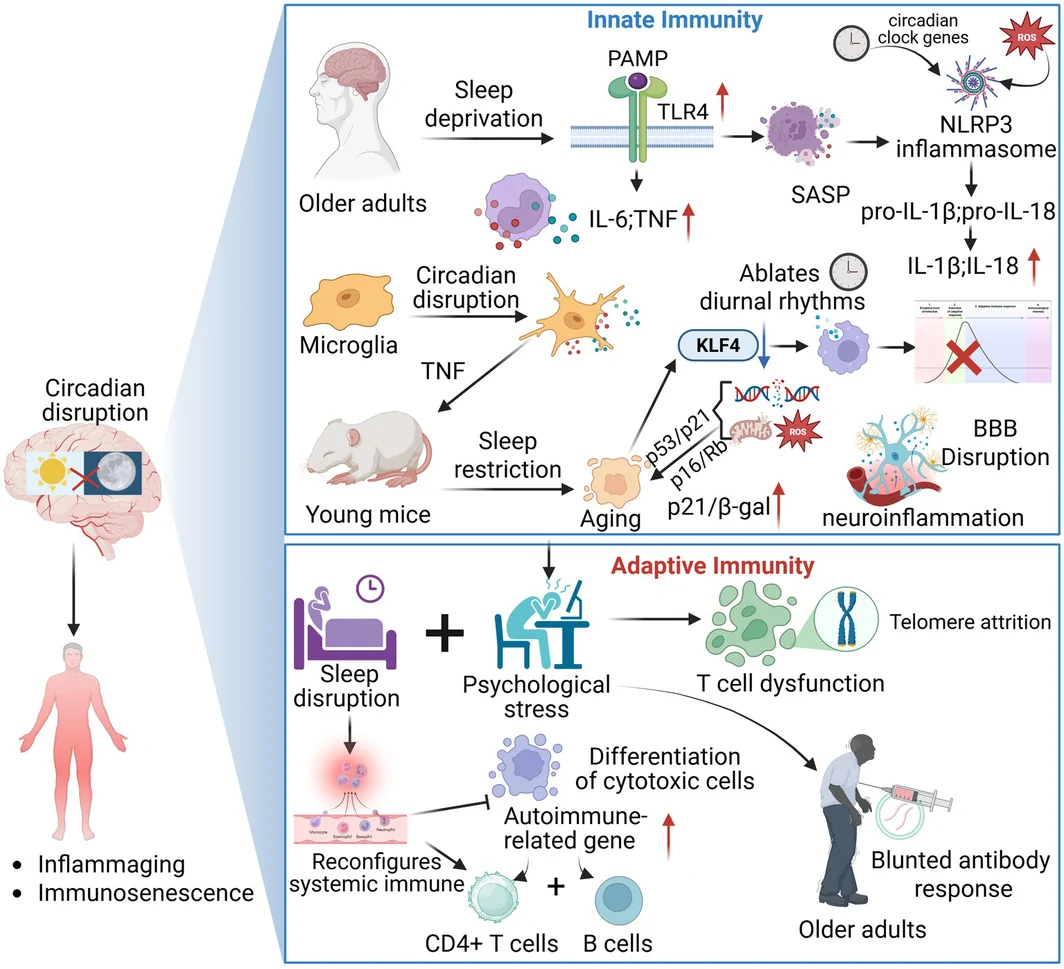
Mechanisms by which sleep disruption exacerbates inflammaging. The figure depicts the hierarchical cascade through which sleep disruption drives immunosenescence and inflammaging. Sleep deprivation and circadian disruption serve as central drivers, concurrently impacting innate and adaptive immunity. Innate Immunity: Sleep deprivation activates TLR4 signaling and NLRP3 inflammasome, promoting pro‐inflammatory cytokines (IL‐6, TNF, IL‐1β, IL‐18) and SASP induction. Sleep restriction in young mice accelerates aging through the p53/p21 and p16/Rb pathways (marked by p21/β‐gal). Furthermore, aging‐induced reduction in KLF4 expression ablates diurnal rhythms in macrophage phagocytosis, driven by diminished rhythmic expression of this transcription factor. These downstream effects collectively culminate in BBB disruption, neuroinflammation, and cellular senescence. Adaptive Immunity: The combination of sleep disruption and psychological stress accelerates T‐cell aging via telomere attrition and T cell dysfunction, leading to a blunted antibody response in older adults. Additionally, sleep disruption reconfigures systemic immunity, influencing the differentiation of cytotoxic cells (upregulating autoimmune‐related genes) as well as the function of CD4^+^ T cells and B cells.

### 3.1 Accelerated Immunosenescence: A Dual Assault on Innate and Adaptive Immunity

Sleep disruption accelerates aging across both arms of the immune system, altering their composition and function.

#### 3.1.1 Innate Immunity: From Hyperactivation to a Senescence Niche

Experimental sleep loss directly provokes pro‐inflammatory responses in an age‐contingent manner. In older adults, partial sleep deprivation amplifies monocytic production of interleukin‐6 (IL‐6) and tumor necrosis factor‐alpha (TNF) via enhanced Toll‐like receptor 4 (TLR4) signaling (Carroll et al. 2015). Even acute sleep restriction activates transcriptional programs linked to the DNA damage response (DDR) and the senescence‐associated secretory phenotype (SASP), directly implicating sleep loss in fundamental aging pathways (Carroll et al. 2016). This link is causal, as sleep restriction in young mice progressively induces cellular senescence markers (e.g., β‐galactosidase, p21) alongside neuroinflammation and blood–brain barrier (BBB) disruption (Avilez‐Avilez et al. 2025).

Aging fundamentally alters the brain's innate immune landscape, creating a primed, hyper‐reactive state. Microglia lose circadian rhythmicity and adopt a tonically elevated phenotype with exaggerated cytokine release upon challenge (Fonken et al. 2016, 2018). This priming is functionally significant: chronic astrocytic TNF production in sleep‐regulatory brain areas suffices to induce aging‐like sleep–wake disturbances in young mice (Kostin et al. 2024). Aged animals also show a critical vulnerability in which sleep fragmentation disrupts BBB integrity and increases TNF transport into the brain, an effect absent in the young (Opp et al. 2015). Similarly, aging ablates diurnal rhythms in macrophage phagocytosis, driven by diminished rhythmic expression of the transcription factor KLF4 (Blacher et al. 2022).

The NLRP3 inflammasome is a key molecular hub integrating circadian disruption, oxidative stress, and innate immune hyperactivation. Regulated by circadian clock genes and reactive oxygen species (ROS), it catalyzes the cleavage of pro‐IL‐1β and pro‐IL‐18 into active, potent inflammatory mediators (Gutowski and Kowalczyk 2013; Hwang et al. 2013; O'Siorain et al. 2024; Bardoulet et al. 2026). OSA provides a powerful clinical paradigm for this pathway, with NLRP3 activation positioned as a therapeutic target (Wei et al. 2025). The systemic consequences of OSA, including premature senescence in visceral adipose tissue, depletion of the anti‐aging hormone klotho, and accelerated telomere attrition, are discussed below as drivers of multisystem inflammaging (Ahmed et al. 2025; Naushad et al. 2024; Pako et al. 2020; Turkiewicz et al. 2021).

Cellular senescence, a stable cell‐cycle arrest driven by DNA damage, oxidative stress, and mitochondrial dysfunction via p53/p21 and p16/Rb, is the mechanistic bridge between sleep loss and sustained inflammation. Senescent cells secrete SASP factors (IL‐6, IL‐1β, TNF, chemokines, proteases) that amplify innate immunity and NLRP3‐driven inflammation. Because sleep loss promotes ROS and DNA damage, it can initiate this senescence‐SASP‐inflammaging loop; senotherapeutics target this node.

#### 3.1.2 Adaptive Immunity: Accelerated T‐Cell Aging and Functional Decline

Sleep disturbance is also a potent accelerator of adaptive immune decline. Converging evidence points to a detrimental synergy in which sleep disruption and psychological stress impair T‐cell integrity, reflected in accelerated telomere attrition (Tempaku et al. 2025; Wolf et al. 2019). This cellular aging manifests as profound functional deficits. Sleep deprivation in humans reconfigures the systemic immune landscape, impairing differentiation and effector potential of cytotoxic cells while upregulating autoimmune‐related gene pathways in CD4^+^ T cells and B cells (Liu et al. 2021). This immunodysregulation may underpin clinical observations linking insomnia symptoms to coagulation activation and heightened cardiovascular risk (Polanka et al. 2021).

The functional consequence is starkly evident in impaired adaptive responses, most notably blunted antibody response to vaccination in older adults. Critically, the detrimental effect of psychological distress on vaccine efficacy is potentiated by co‐existing physiological vulnerabilities such as low body mass index or sedentary behavior (Segerstrom et al. 2012). This age‐related vulnerability is rooted in a fundamental breakdown of circadian immunoregulation. Sleep and circadian disturbances thus exacerbate and accelerate this pre‐existing, age‐related decline in temporal immune organization. Consequently, sleep disruption not only establishes a pro‐inflammatory innate microenvironment through SASP and NLRP3 activation but also simultaneously cripples adaptive immune competence, constituting the complete picture of accelerated immunosenescence. Whether therapeutic sleep improvement can reverse these established deficits, or merely halt their progression, remains unresolved.

### 3.2 Systemic Amplifiers of Inflammation: The Gut–Brain Axis as a Bidirectional Conduit

The immune dysregulation described above is potentiated by systemic pathways, with the gut‐brain axis emerging as a critical amplifier. The core mechanism involves compromised intestinal barrier integrity and dysbiosis following sleep loss, facilitating translocation of microbial products such as lipopolysaccharide (LPS) into systemic circulation and provoking persistent inflammation (Muller and Di Benedetto 2025; Yan et al. 2024). Concurrently, disrupted production of immunomodulatory microbial metabolites (e.g., short‐chain fatty acids) further dysregulates host immune function (Figure 3).

**FIGURE 3:**
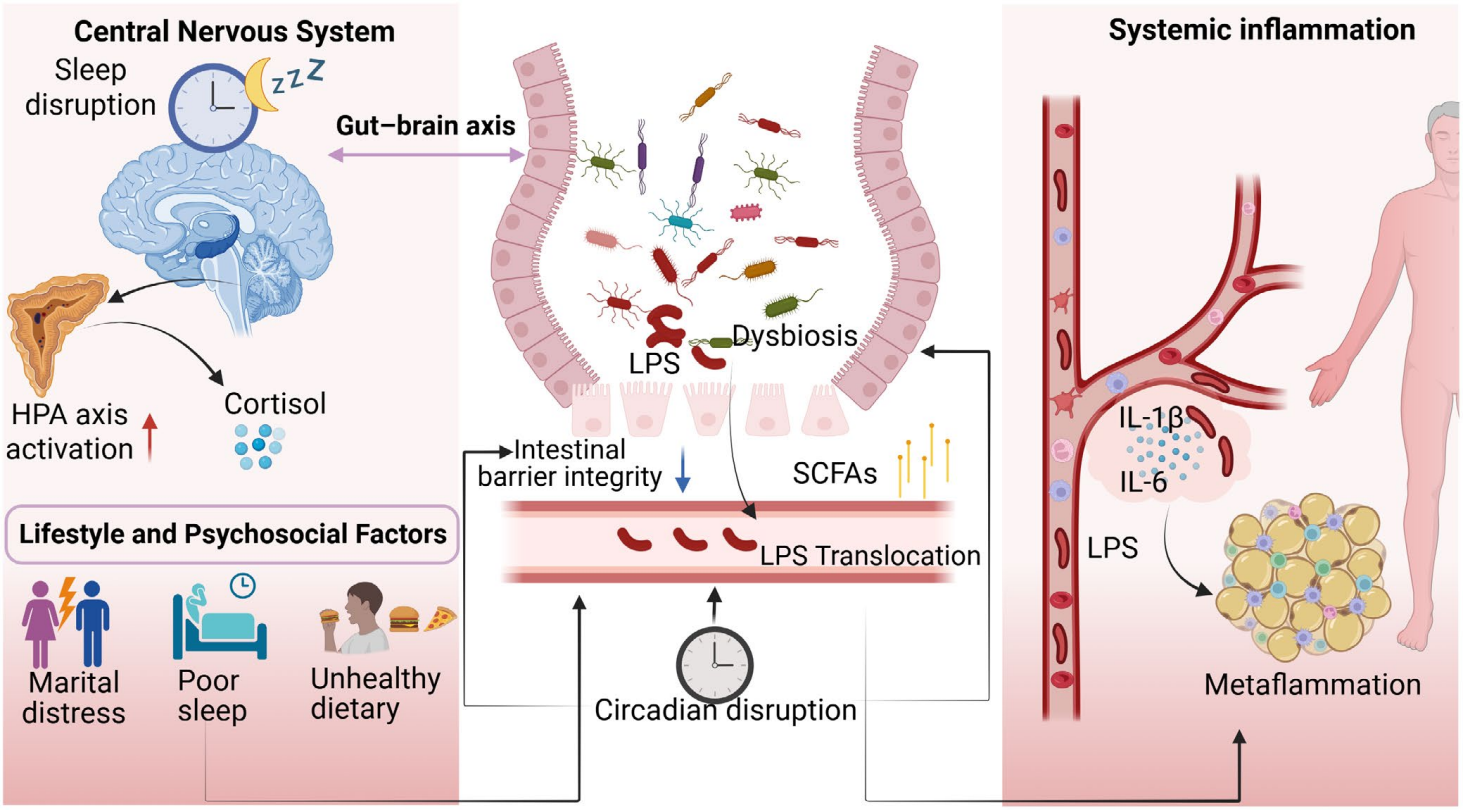
The gut–brain axis as a bidirectional amplifier of sleep‐driven inflammation. The figure illustrates how sleep disruption amplifies systemic inflammation through the gut–brain axis. Sleep disruption activates the HPA axis and sympathetic outflow, transmitting circadian disruption and sleep pressure to the gut. Sleep loss compromises intestinal barrier integrity and induces dysbiosis, leading to LPS translocation and reduced SCFA production. Circadian disruption perturbs gut microbiota rhythms, impairing barrier maintenance. LPS translocation drives systemic inflammation (IL‐1β, IL‐6), which further disrupts circadian clocks, establishing a feed‐forward loop. Lifestyle and psychosocial factors—marital distress, poor sleep, and unhealthy diet—synergistically exacerbate gut dysbiosis and permeability, fueling metaflammation. This gut‐derived low‐grade endotoxemia challenges the central nervous system, highlighting the gut–brain axis as a critical amplifier in sleep‐driven inflammaging.

A crucial interface with circadian biology is the intrinsic diurnal rhythm of the gut microbiota. Microbial compositions and function exhibit robust oscillations synchronized by host cues, including feeding‐fasting cycles and systemic circadian signals (Kiecolt‐Glaser et al. 2019). Sleep disruption disturbs these microbial oscillations, leading to dysbiosis and loss of rhythmic metabolite production (Muller and Di Benedetto 2025). This impairs the circadian timing of intestinal barrier maintenance, creating a permissive environment for endotoxin translocation. The resulting endotoxemia drives systemic inflammation, which can further disrupt central and peripheral circadian clocks, establishing a feed‐forward loop (Franceschi et al. 2018).

The bidirectional nature of this axis is equally important. The brain, through sleep‐ and stress‐induced activation of the hypothalamic–pituitary–adrenal (HPA) axis and sympathetic outflow, directly influences gut permeability, motility, and the microbial ecosystem, translating central circadian disruption and sleep pressure into peripheral inflammatory signals (Kiecolt‐Glaser et al. 2019). Lifestyle and psychosocial factors common in aging converge with sleep disruption to exacerbate this pathway. Marital distress, poor sleep, and unhealthy dietary patterns synergistically promote gut dysbiosis and increase intestinal permeability (leaky gut), fueling systemic inflammation and illustrating a multidirectional relationship between diet, sleep, and immune status (Kiecolt‐Glaser et al. 2019; Evans et al. 2024). These interactions underscore metaflammation, a chronic low‐grade metabolic inflammation sharing core mechanisms with inflammaging (Franceschi et al. 2018).

Sleep disruption does not operate in isolation. The exposome, including Western‐style diet, obesity, physical inactivity, psychosocial stress, socioeconomic disadvantage, and environmental exposures, powerfully modulates metabolic and immune aging. Chronobiological feeding rhythms and diet quality are major determinants of gut barrier integrity, microbial composition, and systemic inflammation. These factors can act synergistically with sleep loss and circadian misalignment and may confound or mediate observed associations. We therefore frame sleep disruption as one important, modifiable node within a broader exposome rather than as a sufficient sole cause of inflammaging.

### 3.3 Central Nervous System Vulnerability: The Collapse of the Glymphatic Clearance System

The resultant systemic inflammatory milieu directly affects the central nervous system. Circulating pro‐inflammatory mediators prime microglia and promote neuroinflammation, forging a deleterious link between peripheral immune dysregulation and brain aging (Daulatzai 2012). Critically, the brain's capacity to clear these mediators and other metabolic wastes is itself sleep‐dependent and profoundly compromised by aging and sleep loss.

The glymphatic system, a brain‐wide perivascular clearance network, is most active during sleep, particularly slow‐wave sleep, and its function declines with age and is impaired by sleep fragmentation (Xiong et al. 2024). Mechanistically, reduced norepinephrine during non‐rapid eye movement (NREM) sleep allows interstitial space expansion, increasing convective exchange between cerebrospinal and interstitial fluid and flushing soluble waste. This process clears not only classical protein aggregates such as amyloid‐beta and hyperphosphorylated tau but also pro‐inflammatory cytokines (e.g., IL‐1β, TNF) and damage‐associated molecular patterns (DAMPs) that fuel neuroinflammation (Xiong et al. 2024).

Sleep disruption therefore delivers a dual hit to the aging brain: it increases production of pro‐inflammatory mediators via pathways such as the gut‐brain axis while concurrently crippling the brain's primary clearance mechanism. This combination of increased load and decreased clearance accelerates cerebral accumulation of damaging proteins and fuels neuroinflammation (Gordleeva et al. 2020; Xiong et al. 2024). Mathematical modeling suggests that beyond a critical inflammaging threshold, this process may become irreversible even upon sleep restoration (Gordleeva et al. 2020). The glymphatic system thus represents a convergent, sleep‐dependent effector mechanism crucial for protecting against cerebral inflammaging and a critical node for therapeutic intervention.

### 3.4 The Master Disruptor: Circadian Dysregulation as a Root Cause

Underlying and connecting all aforementioned mechanisms, from the senescence bias of immune cells to loss of gut barrier integrity to paralysis of the glymphatic system, is the systemic erosion of circadian rhythmicity. The cell‐autonomous molecular clock, governed by core genes such as *BMAL1* and *PER*, is a fundamental regulator of immune homeostasis, driving diurnal oscillations in leukocyte trafficking, metabolism, and cytokine production (Nathan et al. 2021). This precise temporal coordination becomes profoundly degraded with advancing age.

Aging is characterized by systemic erosion of circadian timing. At the organismal level, the master pacemaker in the suprachiasmatic nucleus (SCN) exhibits altered electrophysiology and heightened sensitivity to inflammatory cytokines, indicating that it becomes a direct target of age‐associated inflammation (Bentivoglio et al. 2006). This vulnerability is exacerbated by age‐related reductions in SCN neuronal coupling, diminished sensitivity to photic input, and decreased melatonin receptor expression, all of which lower the central clock's resistance to peripheral inflammatory noise. This central vulnerability is mirrored in peripheral immune tissues. Aged macrophages lose rhythmic expression of core clock genes and key transcription factors like *Klf4*, abolishing diurnal rhythms in phagocytosis (Blacher et al. 2022). Similarly, hippocampal microglia from aged animals display dampened oscillations and tonically elevated pro‐inflammatory cytokine levels, erasing time‐of‐day‐dependent differences in immune reactivity (Fonken et al. 2016).

Experimental models of chronic circadian misalignment directly recapitulate and accelerate these age‐related inflammatory phenotypes. Such paradigms induce chronic low‐grade inflammation, promote immune senescence marked by altered T and B cell subsets, and can even lead to organ pathology and reduced lifespan (de Souza et al. 2025; Inokawa et al. 2020). The mechanisms are multifaceted: circadian disruption primes innate immune cells for hyperresponsiveness (Blacher et al. 2022), exacerbates microglial neuroinflammatory responses (Vallee et al. 2020), and disrupts mitochondrial circadian rhythms, leading to oxidative stress and NLRP3 inflammasome activation, creating a vicious cycle that sustains systemic inflammation (Acuna‐Castroviejo et al. 2017; Lananna and Musiek 2020). Critically, this mechanistic link is supported by human data, in which disrupted rest‐activity rhythms correlate with elevated inflammatory markers and accelerated biological aging (Shim et al. 2023; Xiao et al. 2022).

In summary, circadian disruption is not merely a consequence but a primary driver of inflammaging. The age‐related breakdown of temporal coordination, from the central SCN to tissue‐resident immune cells and systemic barriers such as the gut, sensitizes the organism to inflammatory stimuli, disrupts resolution pathways, and creates a permissive environment for the chronic low‐grade inflammation that defines aging. Consequently, strategies aimed at restoring circadian integrity may represent the most potent intervention to decouple aging from its inflammatory sequelae.

## 4 Clinical and Epidemiological Evidence

### 4.1 Observational Evidence: Establishing Associations

A robust body of clinical and epidemiological data solidifies the association between sleep disturbances and the inflammatory hallmarks of aging (Figure 4). Large‐scale observational studies consistently demonstrate that both poor sleep quality and abnormal sleep duration, including both short and long sleep, are linked to elevated systemic inflammatory markers, such as C‐reactive protein (CRP) and IL‐6 in middle‐aged and older adults (Ballesio et al. 2022; Christian et al. 2011; Jackowska and Steptoe 2015; Irwin 2025; Iakunchykova et al. 2024). This association extends to specific clinical contexts, such as higher IL‐6 levels in individuals with chronic low back pain and sleep disturbance (Heffner et al. 2011; Josefsson et al. 2026), and low testosterone correlating with inflammation in elderly men with metabolic syndrome, independent of sleep duration (Chrysohoou et al. 2013; Rotter et al. 2025). Notably, the link to inflammation appears symptom‐specific: elevated CRP is more strongly associated with neurovegetative depressive symptoms (e.g., fatigue, restless sleep) than with cognitive‐affective symptoms (Chu et al. 2023; White et al. 2017).

**FIGURE 4:**
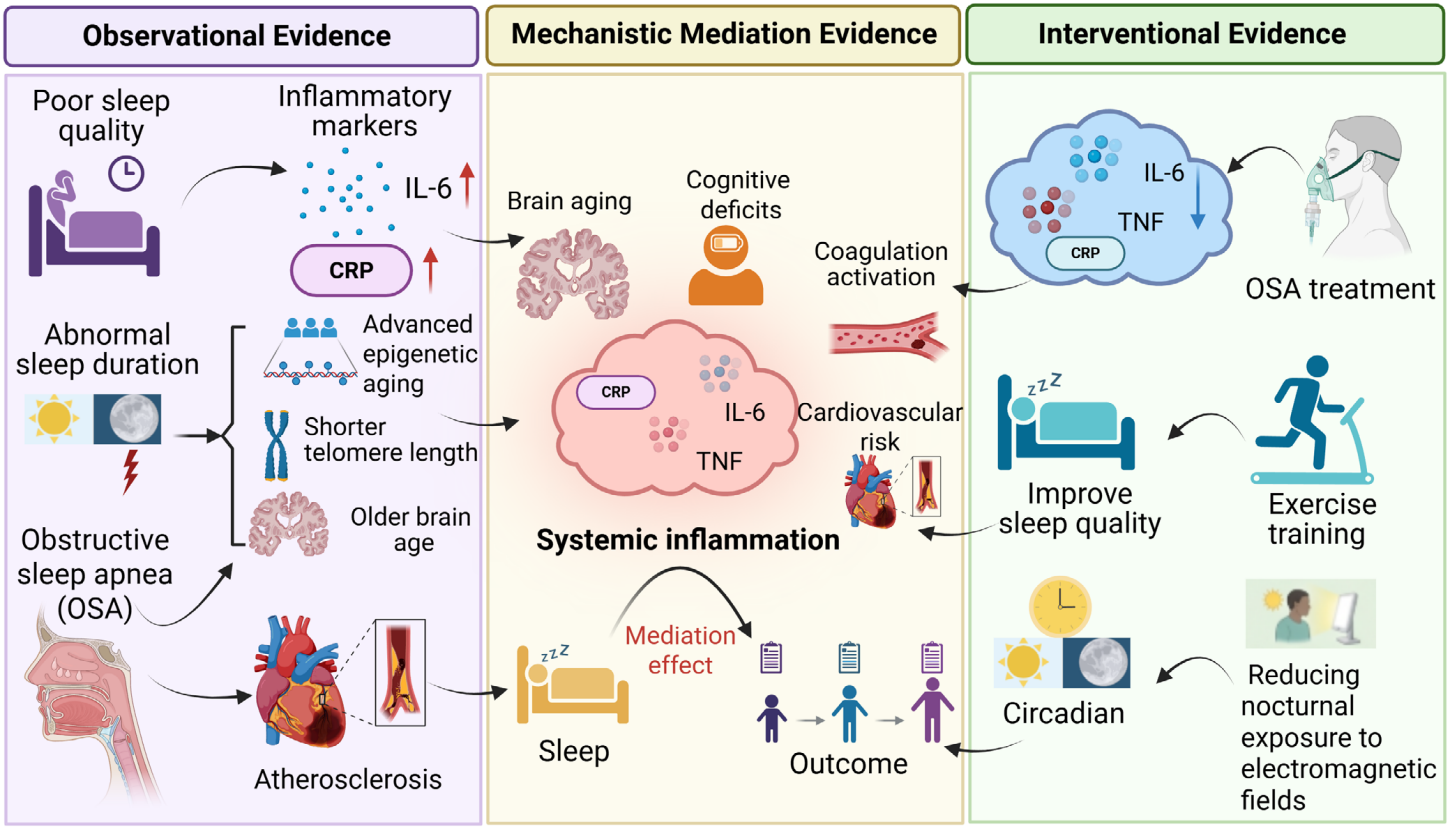
Evidence linking sleep disruption to inflammaging: From observation to intervention. The figure summarizes three levels of evidence supporting the role of sleep disruption in driving inflammaging. Observational evidence shows that poor sleep quality and obstructive sleep apnea (OSA) are associated with elevated inflammatory markers (CRP, IL‐6), accelerated biological aging (epigenetic aging, telomere attrition, older brain age), and atherosclerosis, establishing robust associations between sleep disturbances and inflammaging. Mechanistic mediation evidence identifies systemic inflammation (CRP, IL‐6, TNF) as a mechanistic mediator linking sleep disturbances to adverse outcomes, including brain aging, cognitive deficits, and cardiovascular risk, suggesting causality through inflammatory pathways. Interventional evidence demonstrates that modulating sleep or circadian rhythms through OSA treatment, exercise training, and reducing nocturnal electromagnetic field exposure has shown beneficial effects on inflammatory and aging biomarkers, providing preliminary causal support. Together, these evidence tiers form a coherent framework implicating sleep disruption as a driver of inflammaging.

Sleep disorders, particularly OSA, are strongly implicated in driving inflammaging pathology. OSA severity is associated with increased carotid intima‐media thickness, an atherosclerosis marker linked to inflammation (Chang et al. 2017; Liu et al. 2025), and high OSA risk is a prevalent factor associated with systemic inflammation in large cohorts (Thompson, Legault, Moullec, Baltzan, et al. 2022). Crucially, sleep disturbances are linked not only to inflammatory markers but also to accelerated biological aging processes. Both OSA severity and poor general sleep health are associated with older brain age, advanced epigenetic aging measured by DNA methylation clocks, and shorter leukocyte telomere length, with systemic inflammation identified as a significant mediator (Li et al. 2019; Miao et al. 2025; Riestra et al. 2017; Weihs et al. 2021). These associations translate to clinical risk: long sleep duration independently predicts all‐cause mortality even after accounting for frailty and IL‐6 levels (Lee et al. 2017), while dysregulated diurnal cortisol rhythms predict future functional limitations in older adults (Piazza et al. 2018; Ungvari et al. 2025).

While these observational associations are robust, inferring causality requires caution. The consistent U‐shaped relationship between sleep duration and adverse outcomes may reflect reverse causation, whereby underlying pathology (e.g., chronic inflammation, frailty) drives both long sleep and mortality rather than long sleep itself being harmful. Mendelian randomization (MR) studies using genetic variants associated with sleep traits as instrumental variables are urgently needed to disentangle cause from consequence. Preliminary MR analyses have suggested a causal role for insomnia in depression and cardiovascular disease (Lane et al. 2019; Daghlas et al. 2019; Zhang et al. 2025; Hou et al. 2025), but similar studies for inflammaging endpoints (e.g., epigenetic age acceleration, IL‐6 trajectories) are lacking. Residual confounding by socioeconomic status, physical activity, and comorbid conditions remains a challenge, underscoring the need for triangulation across multiple study designs.

### 4.2 Mechanistic Mediation Evidence: Hinting at Causality

Beyond direct associations, growing evidence identifies systemic inflammation as a mechanistic mediator linking sleep disturbances to adverse aging outcomes. This mediation evidence strengthens the case for causality by demonstrating that the sleep‐outcome relationship operates, at least in part, through inflammatory pathways.

Recent studies using causal inference methods have quantified these mediation effects. Poor sleep health is associated with older brain age, and this relationship is significantly mediated by systemic inflammation (as measured by CRP and IL‐6), suggesting that sleep disruption accelerates brain aging at least partially through inflammatory mechanisms (Miao et al. 2025). Similarly, inflammatory biomarkers partially mediate the association between sleep duration and cognitive function in older adults, with both short and long sleep exerting indirect effects on cognition through elevated inflammation (Wang et al. 2025).

These mediation findings are clinically significant because they identify inflammation as a modifiable intermediate target. If sleep disruption accelerates aging through inflammation, interventions that reduce inflammation, whether directly (e.g., anti‐inflammatory drugs) or indirectly (e.g., diet, exercise), could mitigate downstream consequences of poor sleep even if sleep itself is not fully normalized. This provides a mechanistic rationale for the adjunctive strategies (Tier 2) proposed in Section 5.

The role of inflammation as a mediator extends to specific clinical populations. Thompson and colleagues reported that systemic inflammation partially mediates the association between OSA risk and cognitive deficits in middle‐aged and older adults, implicating inflammatory pathways in the neurocognitive sequelae of sleep‐disordered breathing (Thompson, Legault, Moullec, Martineau‐Dussault, et al. 2022). Insomnia symptoms are linked to coagulation activation, with inflammatory markers playing a mediating role, suggesting a pathway to heightened cardiovascular risk (Polanka et al. 2021).

### 4.3 Interventional Evidence: Testing Causality

Intervention studies, though often preliminary, provide promising evidence that modulating sleep or circadian rhythms can beneficially impact the immune‐inflammatory axis.

Treating OSA with an intraoral appliance reduced oxidative stress and inflammatory markers in the elderly (Peres et al. 2024; Campos‐Rodriguez et al. 2016).

Non‐pharmacological lifestyle interventions show consistent effects: moderate exercise training improved sleep quality while shifting the cytokine profile toward a less inflammatory state (Santos et al. 2012; Korkutata et al. 2025), and various forms of time‐restricted or circadian fasting improved cardiometabolic and inflammatory biomarkers (Alam et al. 2019; Alasmari et al. 2024; Chaix et al. 2021). Respecting circadian biology appears key, as ideal 24‐h physical activity patterns confer greater protective benefits against mortality, an effect mediated in part by attenuated inflammation (Bai et al. 2024). Conversely, severe circadian disruption measured by wearable devices is linked to accelerated biological aging (Shim et al. 2023). Even reducing nocturnal exposure to electromagnetic fields improved immune function and lowered calculated biological age (Diaz‐Del Cerro et al. 2020).

However, most intervention studies have been limited by small sample sizes, short follow‐up durations (typically weeks to months), and heterogeneous outcome measures. The lack of standardized inflammatory endpoints (e.g., consensus on which cytokines and which assays) hinders cross‐study comparability. Furthermore, few studies have examined whether improvements in sleep translate to durable reductions in age‐related disease incidence, the ultimate test of clinical utility. Notably, while CPAP trials have demonstrated improvements in surrogate markers such as blood pressure and inflammatory biomarkers, large‐scale randomized trials have not consistently shown reductions in hard cardiovascular endpoints, highlighting the gap between biomarker changes and clinical outcomes (McEvoy et al. 2016). These limitations underscore the need for the long‐term, endpoint‐driven RCTs advocated in Section 5.2.

### 4.4 Critical Gaps and Future Directions

Despite strong associative evidence, critical questions remain. The consistent U‐shaped relationship between sleep duration and adverse outcomes suggests that long sleep may often reflect underlying pathology rather than directly cause harm, a distinction requiring causal interrogation through Mendelian randomization or carefully designed longitudinal studies. While the cited interventions are promising, most are short‐term; long‐term randomized controlled trials are urgently needed to determine whether sustainably improving sleep quality or circadian alignment can durably slow inflammaging trajectories and reduce the incidence of age‐related diseases. Furthermore, the field must move beyond canonical markers such as CRP and IL‐6 to characterize broader immune cell aging and SASP profiles in relation to sleep architecture. Integrating multi‐omics approaches (e.g., single‐cell transcriptomics, epigenomics, metabolomics) with detailed sleep phenotyping is essential to identify high‐risk immuno‐sleepotypes and enable personalized, preemptive interventions.

Addressing these gaps is essential to move the field from association to clinical action. The prioritization framework proposed in Section 5 (Table 1) represents one attempt to translate current evidence into a structured research and clinical agenda. In this framework, Tier 1 interventions (OSA treatment, CBT‐I [Trauer et al. 2015], circadian reinforcement [Aini et al. 2024; Murukesu et al. 2025]) correspond to the strongest interventional evidence reviewed in Section 4.3; Tier 2 interventions (diet [Lopez‐Gil et al. 2023], exercise [Santos et al. 2012], melatonin for specific subgroups) align with the mediation evidence in Section 4.2, targeting inflammation as a modifiable intermediate; and Tier 3 interventions (senolytics; Shrestha et al. 2023; Hickson et al. 2019, gut microbiome modulation, glymphatic enhancers) highlight areas where the observational associations (Section 4.1) are compelling but interventional evidence remains preliminary, representing the most acute gaps identified here.

**TABLE 1:** A hierarchical approach to sleep interventions: From foundational therapies to emerging strategies.

Priority tier	Intervention category	Examples	Evidence strength	Feasibility	Key considerations	References
Tier 1 (Foundational)	Treatment of primary sleep disorders	OSA management (CPAP, oral appliances)	High (RCTs)	High	First‐line; reimbursed; directly targets hypoxia/fragmentation	Peres et al. (2024); Campos‐Rodriguez et al. (2016); McEvoy et al. (2016)
	Behavioral sleep hygiene	CBT‐I, stimulus control	High (RCTs)	High	Low cost, no adverse effects; sustainable	Trauer et al. (2015)
	Circadian reinforcement	Light therapy, consistent sleep–wake schedule	Moderate‐High	High	Synergistic with other interventions; minimal risk	Aini et al. (2024); Murukesu et al. (2025)
Tier 2 (Adjunctive)	Dietary patterns	Mediterranean diet, time‐restricted feeding	Moderate	Moderate‐High	Requires behavioral adherence; benefits extend beyond sleep	Lopez‐Gil et al. (2023)
	Structured physical activity	Aerobic exercise, resistance training	Moderate	Moderate	Circadian entrainment; systemic anti‐inflammatory effects	Santos et al. (2012); Bai et al. (2024)
	Melatonin supplementation	Low‐dose prolonged‐release formulations	Low‐Moderate	High	Equivocal efficacy in healthy elderly; useful in specific populations (e.g., circadian phase delay)	Brzezinski et al. (2005); Cardinali et al. (2022)
Tier 3 (Emerging/Inv‐estigational)	Senolytics	Dasatinib + quercetin, fisetin, HCW9218	PreclinicTheoretical; no human data yet al/Early Phase	Low (investigational)	Promising but long‐term safety unknown; requires rigorous RCTs	Shrestha et al. (2023); Hickson et al. (2019); Justice et al. (2019)
	Gut microbiome modulation	Probiotics, prebiotics, fecal transplant	Preclinical/Low	Moderate	Mechanistic rationale strong; clinical data in sleep‐aging context sparse	Muller and Di Benedetto (2025); Kiecolt‐Glaser et al. (2019)
	Glymphatic enhancers	Drugs targeting aquaporin‐4, norepinephrine modulation	Preclinical	Low	Theoretical; no human data yet	Xiong et al. (2024); Gordleeva et al. (2020)

## 5 Therapeutic Implications and Future Directions

Targeting the sleep‐immune axis is a promising yet underexplored strategy for mitigating inflammaging and promoting healthy longevity. Disrupting the self‐perpetuating cycle of sleep disturbance and systemic inflammation necessitates multimodal interventions, broadly categorized as pharmacological and non‐pharmacological.

### 5.1 Integrated Intervention Strategies

Pharmacological strategies focus on molecules that entrain circadian rhythms and exert direct immunomodulatory effects. Melatonin is a prime candidate due to its dual chronobiotic and anti‐inflammatory properties. It regulates circadian timing, scavenges free radicals, inhibits the NLRP3 inflammasome, and modulates sirtuin activity, pathways crucial for cellular senescence and metabolism (Cardinali 2021; Hardeland 2019; Jenwitheesuk et al. 2014; Luo et al. 2020). Its potential extends to reducing neuroinflammation and synaptic dysfunction in age‐related models (Bocheva et al. 2024; Wei et al. 2025) and mitigating inflammatory sequelae in conditions such as COVID‐19 (Shneider et al. 2020; Souissi et al. 2025). However, clinical evidence for melatonin's efficacy in healthy older adults remains equivocal, with large‐scale meta‐analyses reporting modest effects on sleep parameters and inconsistent impacts on inflammatory biomarkers, likely due to heterogeneous dosing, formulations, and inter‐individual variability in baseline melatonin secretion (Brzezinski et al. 2005; Cardinali et al. 2022; Mdluli et al. 2025). Nevertheless, melatonin remains valuable for specific subgroups, such as individuals with delayed sleep–wake phase disorder (DSWPD; defined in Section 1.1) or low endogenous melatonin production, where targeted replacement may yield more consistent benefits (Zisapel 2018; Auger et al. 2015). This underscores the need for personalized dosing and longer‐term trials with standardized inflammatory endpoints.

Other agents with emerging potential include thyrotropin‐releasing hormone (TRH), which reduced fatigue and CRP in a small study of cancer survivors, hinting at broader immunomodulation (Kamath 2012), and sirtuin activators such as resveratrol, which share anti‐inflammatory and circadian regulatory pathways with caloric restriction (Ramis et al. 2015; Kapar et al. 2024).

Novel senotherapeutics represent a frontier in targeting the sleep‐inflammaging cycle. The immunotherapeutic HCW9218, for example, reduces senescent cell burden and SASP and even restores youthful circadian gene expression patterns in aged mice (Shrestha et al. 2023). More recently, first‐in‐human trials of senolytics (e.g., dasatinib plus quercetin, fisetin) have demonstrated feasibility in reducing senescent cell markers in various age‐related conditions, including idiopathic pulmonary fibrosis and diabetic kidney disease (Hickson et al. 2019; Justice et al. 2019). These emerging data position senolytics as potential dual‐action therapy targeting both the cellular drivers of aging and the sleep disturbances that perpetuate inflammaging.

### 5.2 Prioritizing Interventions: A Framework Based on Evidence and Feasibility

Given the expanding landscape of potential interventions, it is critical to prioritize strategies based on current evidence strength, feasibility, and safety in older populations. Based on the evidence reviewed in Sections 4 and 5.1, we propose the following prioritization framework (Table 1) to guide clinical decision‐making and future research agendas.

This framework underscores that foundational interventions, treating underlying sleep disorders and reinforcing circadian hygiene, should be the first line of defense given their robust evidence base and high feasibility. Adjunctive strategies can amplify benefits and target residual inflammation. Emerging interventions, while mechanistically exciting, require rigorous validation before clinical deployment. Importantly, these tiers are not mutually exclusive; multimodal approaches combining interventions across tiers are likely to yield the greatest impact on the sleep‐inflammaging cycle.

Future research should focus on developing adaptive multimodal interventions that combine foundational, adjunctive, and emerging strategies in a personalized, stage‐dependent manner. Such an approach would allow for dynamic adjustment of treatment intensity and composition based on individual response trajectories, inflammatory biomarkers, and sleep phenotyping, ultimately moving toward precision sleep medicine for healthy aging.

### 5.3 Future Directions and Unresolved Questions

Critical translational gaps must be addressed to advance this field from association to actionable clinical strategy.

#### 5.3.1 Establishing Causality via Rigorous Trials

While epidemiological links are robust, definitive proof requires long‐term, randomized controlled trials (RCTs) testing whether improving sleep and circadian alignment directly decelerates immunosenescence and reduces age‐related disease incidence (Irwin and Opp 2017). Future RCTs should: (a) Focus on midlife to older adults (e.g., 50–75 years) with modifiable sleep disturbances but without advanced comorbidities; (b) Test structured, multi‐modal interventions (e.g., combining cognitive‐behavioral therapy for insomnia, circadian reinforcement, and OSA treatment) against appropriate controls over sufficient durations (e.g., 6–12 months); (c) Employ composite indices of biological aging (e.g., epigenetic clocks, immune cell aging metrics, functional immunocompetence) as primary endpoints, moving beyond standard inflammatory markers; and (d) Incorporate long‐term follow‐up (2–5 years) to assess sustainability and impact on clinical endpoints like cognitive decline or cardiovascular events.

#### 5.3.2 Toward Personalized Sleep‐Immune Medicine

Future approaches must leverage deep phenotyping to move from population‐level recommendations to individualized strategies. This requires longitudinal multi‐omics profiling (e.g., single‐cell transcriptomics, metabolomics, proteomics) integrated with detailed sleep metrics. Such high‐dimensional data can delineate precise immune cell subsets and circulating mediators most sensitive to sleep loss (Liu et al. 2021). Machine learning can then identify distinct immuno‐sleepotypes, clusters of individuals sharing similar immune responses to sleep disturbance. Validated predictive biomarkers (e.g., microRNA signatures, baseline epigenetic age acceleration) could stratify individuals into risk phenotypes, enabling targeted, preemptive interventions (Forster et al. 2024; Weiss 2025). For instance, an individual with a “pro‐senescent” phenotype might be prioritized for senolytic adjuvant therapy alongside sleep improvement.

#### 5.3.3 Deepening Mechanistic Understanding

Critical mechanistic questions remain. The precise molecular links between specific sleep architecture features (e.g., loss of slow‐wave sleep) and immune cell aging (e.g., T‐cell exhaustion, myeloid polarization) require deeper exploration. The role of the gut‐brain axis and microbial metabolites in sleep‐related inflammaging also warrants further study to identify novel therapeutic targets.

#### 5.3.4 Bridging the Translational Gap

Most mechanistic insights derive from animal models. Bridging these findings to human aging, with its inherent complexity, comorbidities, and psychosocial dimensions, remains a paramount challenge. Future research must prioritize human studies that validate preclinical mechanisms and test integrated, real‐world interventions capable of breaking the vicious cycle of sleep disruption and inflammaging.

## 6 Conclusion

The interplay between sleep disruption and inflammaging constitutes a bidirectional, self‐reinforcing cycle that accelerates immunological decline and functional deterioration in aging. Sleep disruption, when chronic, acts as an active driver of immunological aging in susceptible individuals. This review has synthesized evidence demonstrating that sleep is an active period of immune regulation, and its fragmentation, alongside circadian misalignment, drives innate immune hyperactivation (via NLRP3 inflammasome and TLR4 signaling), promotes cellular senescence and the SASP, impairs adaptive immunity (T‐cell aging and telomere attrition), and disrupts systemic barriers such as the gut‐brain axis and the glymphatic clearance system.

Clinical evidence consistently associates poor sleep with elevated inflammatory markers, epigenetic aging, and increased risk of age‐related diseases. Importantly, sleep disturbances are not merely consequences of aging but active contributors to the inflammatory cascade, reaching a threshold beyond which mathematical modeling suggests reversibility may be limited (Gordleeva et al. 2020). The identification of inflammation as a mechanistic mediator linking sleep to cognitive decline (Miao et al. 2025; Wang et al. 2025) and biological aging further strengthens the case for causality and highlights modifiable intermediate targets.

Therapeutic strategies targeting the sleep‐immune axis, including pharmacological agents such as melatonin (with attention to specific subgroups), emerging senotherapeutics supported by recent data (Shrestha et al. 2023), and non‐pharmacological interventions such as circadian alignment, dietary modification, and treatment of sleep disorders, offer promising avenues to decouple aging from its inflammatory sequelae. The prioritization framework proposed in this review (Table 1) provides a structured approach to translating current evidence into clinical action, with Tier 1 interventions (OSA treatment, CBT‐I, circadian reinforcement) representing the strongest evidence base, and Tier 3 interventions highlighting areas where mechanistic promise meets urgent research needs.

Future research must prioritize long‐term randomized controlled trials to establish causality, integrate multi‐omics approaches for personalized sleep‐immune profiling, and deepen mechanistic understanding of how specific sleep features modulate immune aging. The path forward lies in precision sleep medicine: the integration of deep phenotyping (sleep architecture, circadian rhythms, immune cell subsets, inflammatory biomarkers, SASP factors) with adaptive, multimodal interventions tailored to individual trajectories and stage of immunological aging (Nathan et al. 2021; Muller and Di Benedetto 2025). Such an approach would allow for dynamic adjustment of treatment intensity and composition based on individual response patterns, moving beyond one‐size‐fits‐all recommendations toward truly personalized intervention strategies.

Ultimately, preserving sleep and circadian integrity may prove essential for healthy aging and reducing the burden of age‐related chronic diseases. In the quest for healthy longevity, perhaps the most accessible fountain of youth lies not in a pill, but in the nightly restoration of a good night's sleep, a biological process that, when optimized, synchronizes the immune system, clears the aging brain, and resets the circadian clock that orchestrates it all.

## Author Contributions

H.‐Q.W. designed the framework of this article and drafted the manuscript. F.‐Y.Z. created the figures. S.‐Y.L. and J.‐M.W. illustrated the figures; L.H. and H.‐Y.Z. critically revised the manuscript for intellectual content and ensured scientific rigor. All authors contributed to the article and approved the submitted version.

## Funding

This work was supported by the Doctoral Start‐up Foundation of Liaoning Province (no. 2025‐BS‐0627) and Natural Science Foundation of Liaoning Province (no. 2024‐MSLH‐542).

## Conflicts of Interest

The authors declare no conflicts of interest.

## Data Availability

Data sharing is not applicable to this article as no datasets were generated or analyzed during the current study.
